# Evaluation of macular retinal oximetry across different levels of diabetic retinopathy: a cross sectional study

**DOI:** 10.1186/s12886-025-03850-1

**Published:** 2025-01-17

**Authors:** Jennyffer D. Smith, Kaitlyn A. Sapoznik, Kelly Bisignano, Julia Benoit, Wendy W. Harrison

**Affiliations:** 1 College of Optometry, University of Houston College of Optometry, 4401 Martin Luther King Blvd, 77204-2020 Houston, TX USA; 2 Texas Institute for Measurement, Evaluation, and Statistics, 4349 Martin Luther King Blvd, Houston, 77204-6022 TX USA

**Keywords:** Diabetes, Diabetic retinopathy, Optical coherence tomography angiography, Oxygen saturation

## Abstract

**Background:**

This study evaluates retinal oxygen saturation and vessel density within the macula and correlates these measures in controls and subjects with type 2 diabetes (DM) with (DMR) and without (DMnR) retinopathy. Changes in retinal oxygen saturation have not been evaluated regionally in diabetic patients.

**Methods:**

Data from seventy subjects (28 controls, 26 DMnR, and 16 DMR were analyzed. For those with DMR,8 were mild/moderate diabetic retinopathy (NPDR) and 8 severe NPDR/proliferative (PDR). Subjects were categorized with glycosylated hemoglobin A1c and fundus photography. Retinal oximetry measurements were performed within a 300–400 μm region at four diagonal locations 3.1 degrees from the center of the fovea in the superior nasal, superior temporal, inferior nasal, and inferior temporal locations adjacent to the foveal avascular zone (FAZ). Optical coherence tomography angiography (OCTA) was performed and corrected for refractive error. Photoshop and ImageJ were utilized to calculate the superficial capillary plexus vascular density (SCP). Oximetry and OCTA vessel density were analyzed overall and by region.

**Results:**

Average retinal oxygen saturation was highest in DMR (*p* = 0.008). Average OCTA density was less in DMR compared to controls (*p* = 0.01), but not compared to DMnR subjects (*p* = 0.07). A significant inverse correlation was observed between averaged oxygen saturation and SCP vascular density for all subjects (*p* = 0.02). Duration of DM was positively associated with oxygen saturation (*p* = 0.01) and negatively with OCTA SCP vascular density (*p* = 0.009). There were no differential effects of retinal location.

**Conclusion:**

To our knowledge, this study is the first to evaluate the relationship between macular oxygen saturation and SCP vascular density at different levels of retinopathy. This may be useful to track patients with DM as they move through stages of retinopathy.

## Introduction

Diabetic retinopathy (DR) is a leading cause of preventable blindness in working aged Americans [1]. It is characterized by vascular and neuronal changes that can lead to inflammation, oxidative stress, and retinal ischemia [2]. These structural and functional changes significantly affect the metabolic demand and activity in the retina. Retinal oxygen saturation has been evaluated as a metabolic biomarker for the retina that is altered in DR. In non-proliferative DR (NPDR), oxygen saturation in the larger vessels is positively correlated with increasing severity of DR [3–5]. However, in proliferative DR, oxygen saturation may continue to increase or decline [5, 6]. The initial climb in oxygen saturation is thought to be the result of a compensatory mechanism in response to the increase in ischemic changes that occur as DR progresses [7–9].

Retinal oximetry provides a noninvasive measurement of oxygen saturation within the retinal vasculature [10–12]. At present, commercially available retinal oximeters (e.g. the Oxymap T1, Oxymap Inc., Reykjavik, Iceland) exist, however, they are limited in that they are only capable of generating oxygen saturation measurements within the larger caliber vessels of the inner retina adjacent to the optic nerve head. They find values ranging from 70% in the venous vessels and 90% in the arteriole vessels [13]. Detailed methods used in commercial instruments have been described in detail elsewhere [14–17]. Briefly, these instruments work by capturing two simultaneous fundus images with two different wavelengths: one that is oxygen sensitive and the other that is oxygen insensitive, with a ratio computed that represents a linear relationship with oxygen saturation [11]. The study of oxygen saturation within the retina allows for evaluation of the metabolic demand in both healthy and pathological states. One of these disease states where oxygen saturation changes is DR. Disrupted levels of oxygen saturation to the retinal tissues within the process of DR can contribute to advanced vascular and neuronal changes, leading to vision loss.

Most of the analyses performed thus far on retinal oxygen saturation in diabetes have been limited to capturing oximetry measures in only the larger caliber vessels with the technology described above [14–16]. It is not known if the same relationships with retinal oxygen saturation and retinopathy grade hold true for that of the microvasculature near the macula. Oxygen exchange is a principal function of the capillary beds of the retina [10]. Thus, alterations in oxygen saturation at these sites of oxygen transport due to DR could have significant effects on vascular and neuronal health. Recent advances in retinal oximetry techniques enable retinal oxygen saturation measurements within both the superficial and deep capillary beds collectively using the Zilia Ocular Fundus Camera (Zilia Inc., Quebec, Canada). To compute oxygen saturation within the capillary beds of the macula and adjacent tissue, this instrument uses diffuse reflectance spectroscopy. White light emitting diodes enter the eye, are collected within a 300–400 μm movable region, and are reflected from the fundus back to the spectrometer. The instrument’s proprietary algorithms analyze the absorption spectra of oxygenated and deoxygenated hemoglobin and report a mean value. Oxygen saturation measurements are displayed between the range of 0 -100%. Reported oxygen saturation is the percent of hemoglobin in this tissue that is currently bound to oxygen [17]. Detailed principles of this technology are described elsewhere [18–20]. Repeatability studies show the expected measures of larger arteries and venules [21], a diurnal variation in oxygen saturation in young controls [22], and phantom tissue and animal models [23–25] that have collectively begun to validate this novel technique. In this study, we measure the retinal macular oxygen saturation of the capillary beds within the parafovea, just outside the foveal avascular zone in different levels of retinopathy. In addition, we compare oxygen saturation to the retinal vascular density in that area. To our knowledge, this is the first study to evaluate oxygen saturation values within the macula and at the capillary level in different stages of diabetic retinopathy.

## Methods

### Subject demographics

A total of 75 subjects were recruited from the family practice service at the University of Houston College of Optometry during their comprehensive eye examination (09/2021-12/2023). The study visit took place immediately after their eye examination, so all subjects presented to the study with dilated pupils. In each subject, glycosylated hemoglobin (HbA1c) was measured (DCA Vantage Analyzer, Seimens Healthineers) with a fingerpick from the subject’s preferred finger. Subjects were categorized as either control subjects or subjects with diabetes based on their HbA1C reading at their study visit and/or in addition to a previous diabetes diagnosis. Those with diabetes self-reported duration of disease. Exclusion criteria included pregnancy, autoimmune disease, non-diabetic related eye diseases (e.g. macular degeneration), and any previous retinal surgery including lasers for diabetic retinopathy. Potential subjects were screened for confounding ocular/systemic conditions by reviewing, with the individual’s permission, existing eye exam records, when applicable. An effort was made to recruit control and diabetic subjects in a similar age range and demographic for each group. All subjects provided written consent, as well as access to their eye exam from that day. Informed consent was obtained from all subjects. This study was approved by and in accordance with regulations set by the Institutional Review Board of the University of Houston and complied with the tenants of the Declaration of Helsinki.

### Oximetry

Retinal oximetry was performed on the right eye of each participant using an oximeter/fundus camera (Zilia Ocular, Zilia Inc., Quebec, Canada). In each subject, measurements (300–400 μm diameter/1.5 degree acquisition region) were acquired in 10 s intervals capturing an average of 20 readings within that area that were then averaged together by the automated system of the instrument. A mechanism is integrated in the device to optically compensate for the subject’s refractive error and axial length which accounts for a range of -9.50D to + 17.54D. The acquisition region is thus maintained at 1.5 degrees for this refractive error range. Acquisitions were captured at four regions equidistant to the fovea: 3.1 degrees in the superior temporal, inferior temporal, superior nasal, and inferior nasal locations by placing the acquisition region at specified locations on a grid superimposed on the fundus image. Caution was taken to avoid acquisition over medium or large sized vessels (~ 151 μm). Subjects were instructed to fixate on an internal central blue fixation target to minimize eye movements. The device calculates the movements of the eye and data points where eye movements are greater than the radius of the acquisition region were automatically discarded by the instrument. The examiner also removed data points in the rare instance where an eye movement caused them to fall on a medium or larger sized blood vessel.

### Optical coherence tomography angiography

OCT-angiography (OCTA) 6 × 6 mm macula centered scans were obtained on the right eye of each subject (Zeiss Cirrus OCT, Carl Zeiss Meditec, Dublin, CA, USA). Vessel density and foveal avascular zone (FAZ) size for each subject were collected. OCTA image slabs were analyzed using the superficial capillary plexus (SCP) only due to ease of analysis and image quality. SCP is defined as the vasculature contained within the inner plexiform layer to the nerve fiber layer [26], while vessel density for this study was defined as the percent of vasculature within a retinal area with active blood flow. OCTA images were exported from the instrument and vessel density was computed for whole images and regionally cropped images using (Photoshop CC, Adobe System, San Jose, CA) and ImageJ (National Institutes of Health, Bethesda, MD, USA). Procedures for regional data acquisition provided below. The correction factor below was applied to whole images to account for refractive error:

Retinal magnification (correction factor) = ((AL-24) + 16.53)/(16.53), where AL is the average axial length of 24 mm and 16.53 is the distance from the posterior lens capsule (N) to the retinal focal point (F’) as represented in the Indiana eye model.[27].

### Fundus photography

A 133-degree fundus photograph (Zeiss Clarus 500, Carl Zeiss Meditc, Double, CA, USA) was acquired for each subject. Fundus photos were used to assess the presence and grade of DR and ensure no additional retinal pathology was present. For those with retinopathy, a grader masked to subject identity, demographics, and clinical data (KAS) graded fundus photographs using the ETDRS grading system [28]. Additional information about the subject’s duration of diabetes, and other health factors was recorded from the medical chart of their eye exam visit with their permission.

### Regional Data Acquisition

**Fig. 1:**
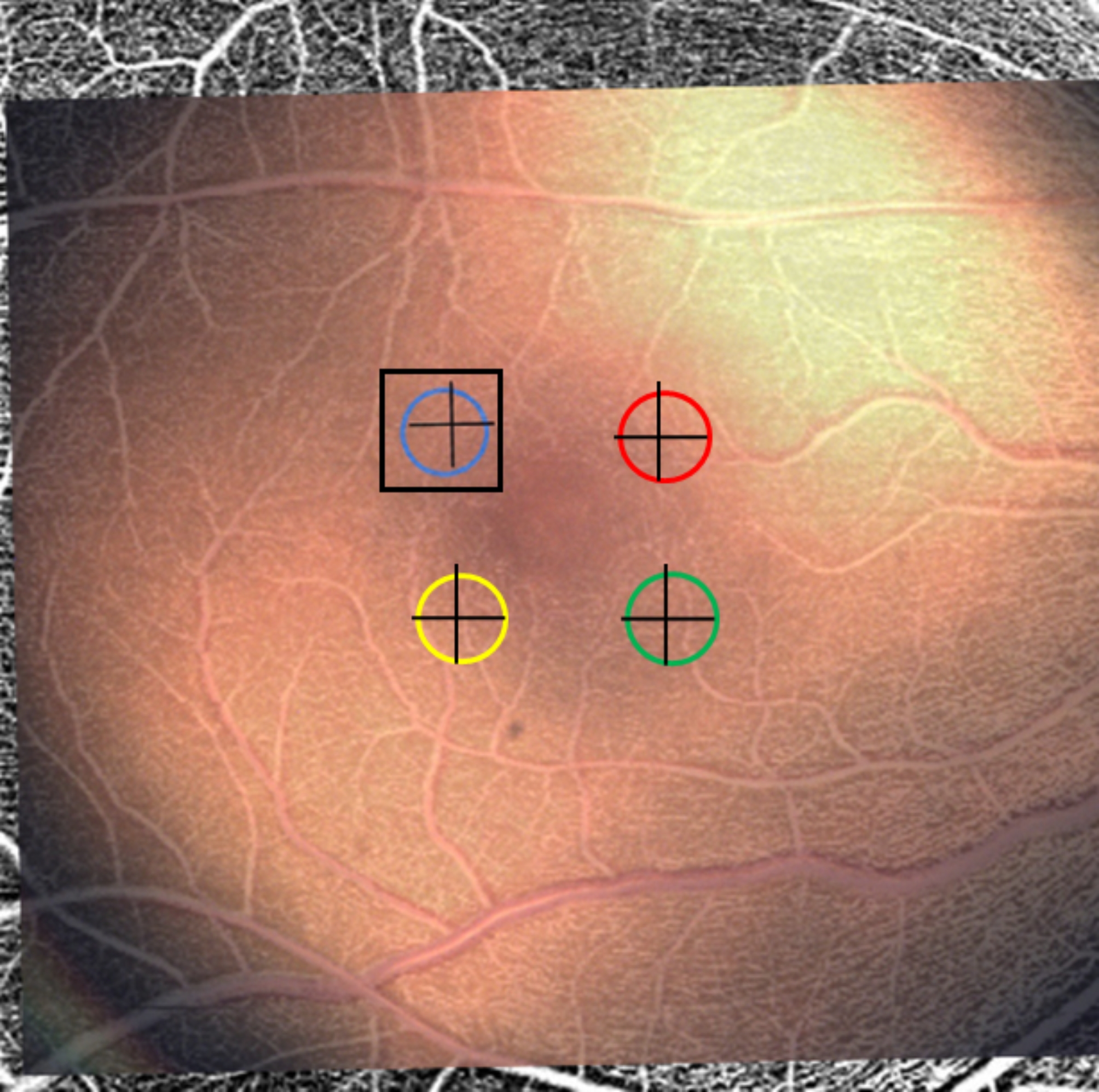
OCTA and oximetry image overlay. Superimposition of OCTA image under oximetry fundus image. Colored circles show acquisition regions of oximetry at the four locations. OCTA regions were matched and cropped as shown by the example box for the superior/temporal location

In addition to the average oxygen saturation readings per target location, whole images of the fundus during measurement acquisition are available for export from the Zilia retinal oximeter. For each subject, four images were acquired as representative samples respectively containing each of the four region retinal locations. Each of the oximetry fundus photos were aligned repeatedly with that subject’s SCP OCTA image using Photoshop. During alignment, vessels were used to align the oximetry fundus image on top of the OCTA image (Fig. 1). A 0.50 mm x 0.50 mm square was cropped through both images enclosing the 300–400 μm circular oximetry retinal region. Alignment was done by the same examiner (JDS) by hand on magnified images for all regions ensuring proper alignment of each image. The cropped OCTA images were converted to 32-bit image types and binarized. Vessel density was computed with ImageJ, then converted from pixels to percentage, which is the unit of measure used when describing the results.

### Statistics

To evaluate the primary aim of this study, oxygen saturation was averaged across the four eye locations acquired for each subject. Data was ensured normal both graphically and with the Shapiro-Wilk test. OCTA vessel density was averaged for the four cropped retinal areas correlating to the four oximetry locations. Averaged four region oxygen saturation was compared between controls, subjects with DM, but without retinopathy (DMnR), and subjects with DM with retinopathy (DMR) groups using corrected unpaired Student’s Ttests and ANOVA with post hoc analysis. OCTA vessel density comparisons between groups were done similarly, however, of note, a one-tailed T-test was used for OCTA group comparisons due to the one-directional change in vessel density when diabetes progresses. With increased retinopathy, density only decreases. Relationships between OCTA vessel density and oxygen saturation were evaluated through multiple regression analysis which controlled for previously determined cofounders such as age and race [28]. These were also performed to compare oxygen saturation and duration of diabetic disease. A mixed model repeated measures analysis (STATA 18) was also performed and assessed whether OCTA vessel density differed by eye location, by group, and whether location effects differed by group. Specifically, we tested for effects of within-eye location (temporal v nasal; inferior v. superior), between-subject factor (Control, DMnR, DMR) groups, and location by group interactions. This approach was taken to evaluate location effects of oxygen saturation as well.

## Results

### Subjects

Five subjects were not included in the final analysis due to unsuccessful data acquisition on the oximeter. Seventy subjects were included in the final analysis and consisted of 28 healthy controls, 26 DMnR, and 16 DMR. Those with retinopathy were further categorized into 8 subjects with mild or moderate nonproliferative diabetic retinopathy (NPDR) and 8 subjects with severe NPDR or proliferative diabetic retinopathy (PDR). All subjects were 30–70 years of age (average 52.4 ± 9.9). Age was not statistically different between groups (*p* = 0.07). All groups were racially diverse and similar, and pigment differences were controlled for in oximetry calculations we have shown elsewhere that oximetry has variations by race in our data.

### Oxygen Saturation comparison by Group

**Fig. 2:**
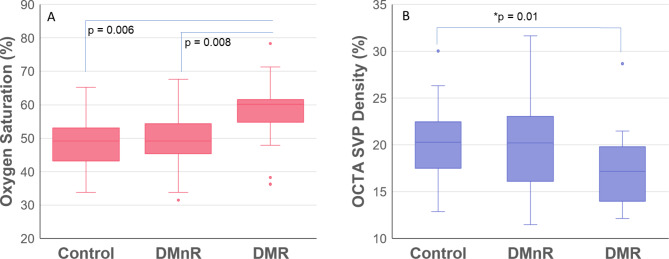
The relationship between oxygen saturation (**A**) and OCTA (**B**) across groups

A summary of the subjects included in the final analysis, including oximetry and OCTA outcome are provided in Table 1. Averaged oxygen saturation was significantly higher in DMR compared to control and DMnR groups (*p* = 0.006 and *p* = 0.008, respectively, Fig. 2A). Overall, the severe NPDR/PDR group of DMR had significantly higher oxygen saturation values relative to the control (*p* = 0.013) and DMnR groups (*p* = 0.001), respectively. The same result was found for the regionally analyzed data by group. For disease group differences, the severe NPDR/PDR group had significantly higher O2 values relative to the control (least square mean: 6.74; standard error: 1.89; *p* = 0.013) and DMnR groups (least square mean: 5.76; standard error: 1.92; *p* = 0.001), respectively.

**Table 1:** Subject demographics

Subject Variables	Control (*n* = 28)	DMnR (*n* = 26)	DMR (*n* = 16)	*P* values
Mean Age ± SD	48.7 ± 10.6	55.2 ± 8.5	54.3 ± 9.8	0.07
Female, n (%)	16 (57.1%)	13 (50%)	5 (31.2%)	
†Race/Ethnicity, n (%)				
White	3 (10.7%)	6 (23.1%)	2 (12.5%)	
Black	10 (35.7%)	9 (34.6%)	3 (18.8%)	
Hispanic	10 (35.7%)	9 (34.6%)	4 (25%)	
Asian	3 (10.7%)	1 (3.8%)	3 (18.8%)	
Other	2 (7.1%)	1 (3.8%)	4 (25%)	
Mean HbA1c ± SD	5.6 ± 0.4	7.4 ± 1.5	8.3 ± 2.2	< 0.01
Mean Duration (years) ± SD	---	8.2 ± 6.2	12.8 ± 8.5	0.01
Mean Oximetry (%) ± SD	48.6 ± 8.4	48.9 ± 8.8	57.9 ± 10.9	< 0.01
Mean OCTA (%) ± SD	20.1 ± 3.8	20.0 ± 4.5	17.5 ± 4.1	0.44

### Vessel density and Oxygen Saturation

**Fig. 3:**
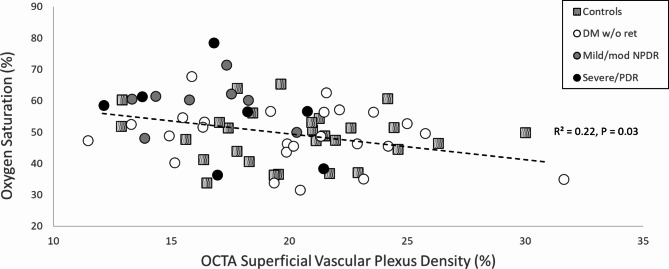
Relationship between oxygen saturation and OCTA vessel density

OCTA vessel density was significantly lower in the DMR group compared to controls (*p* = 0.01, one-tailed t-test, Fig. 2b), but not compared to the DMnR group. A significant correlation was found between OCTA vessel density and oxygen saturation with all subjects together (r² = 0.22, *p* = 0.03, Fig. 3). In the multivariate model for saturation vs. vascular density the coefficient was − 0.01 for vascular density (*p* = 0.029), -1.12 for race (*p* = 0.058) and 0.323 for age (*p* = 0.004), Table 2. Foveal avascular zone size did not stay in the model during analysis.

**Table 2:** Multivariate regression: oxygen saturation (all subjects)

Interaction Variable	Coefficient	*P*-value
Vascular Density	-0.01	0.029
Race	-1.12	0.058
Age	0.323	0.004

### Effect of duration of diabetes

**Fig. 4:**
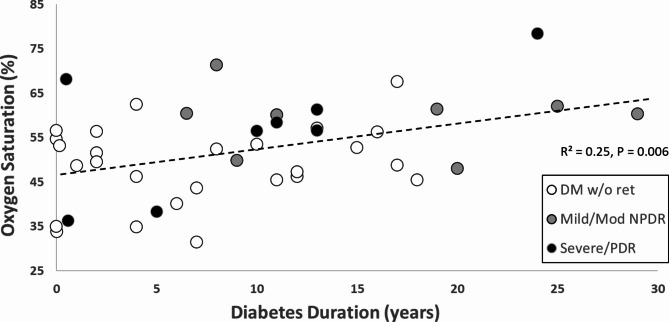
Relationship between oxygen saturation and diabetes duration

In our subjects with DM, a positive correlation was found between oxygen saturation and duration of diabetes in years (controlled for race and age, *p* = 0.006, r^2^ = 0.25, Fig. 4). Coefficients for duration, race and age were 0.42, -0.95 and 0.24 respectively. Race was a confounder with a p value = 0.11, but age was also a significant factor, *p* = 0.038, Table 3. As the years of diabetes increased, oxygen saturation increased for both those with and without retinopathy overall.

**Table 3:** Multivariate regression: oxygen saturation in diabetes

Factor	Coefficient	*P*-value
Duration	0.42	0.006
Race	-0.95	0.11
Age	0.24	0.038

### Additional regional data

**Fig. 5:**
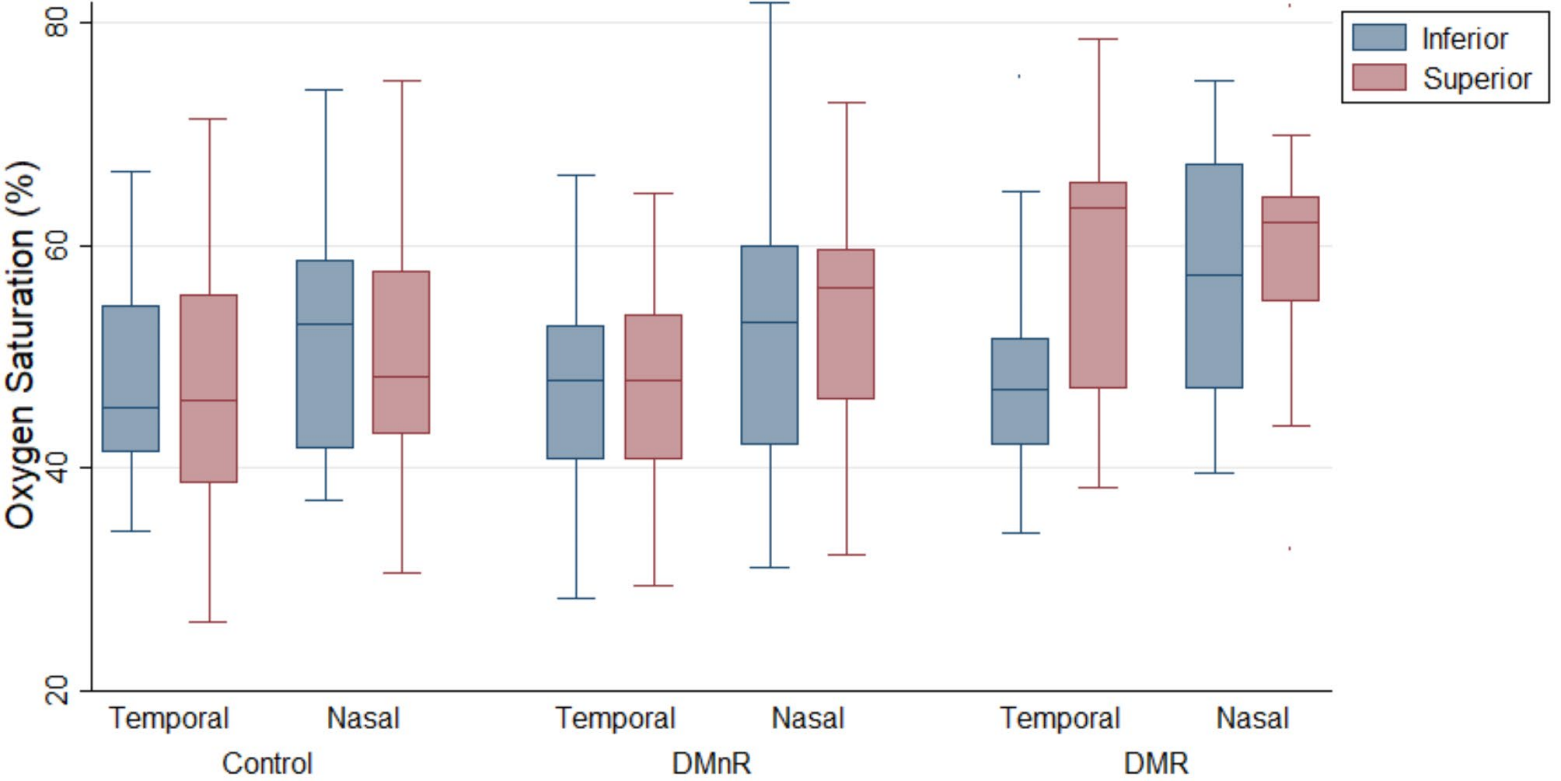
Oxygen saturation differences by location compared between controls, DMnR, and DMR

There were no differential effects of location on group differences (i.e., no statistically significant interaction terms). On average, nasal oxygen saturation was significantly higher than temporal oxygen saturation for all subjects (mean difference: 4.35; standard error: 1.47; *p* = 0.003). This was a fixed effect that did not vary by disease status group. The magnitude of difference between nasal and temporal locations trended larger in the most severely diseased DMR group, however, did not reach statistical significance. Additionally for vessel density, the nasal location resulted in significantly higher OCTA vessel density relative to temporal location (mean difference: 312.18; standard error: 134.36; *p* = 0.021). See Fig. 5 for all oximetry location comparisons.

## Discussion

The present study demonstrates oxygen saturation can be measured in the macula at the capillary level in DR. We show that increases in oxygen saturation within the superficial capillary plexus follow that of diabetic disease pathology within the larger vasculature as shown in the literature [8, 29, 30]. There are several hypotheses for this increase in oxygen saturation. First, increased oxygen saturation may be due to increased blood flow in the early stages of retinopathy to reoxygenate compromised areas of the retina [32–34]. Second, as a result of nonperfusion and capillary drop out preventing oxygen extraction from the capillary beds and prevention of exchange and exit of oxygen from venous passageways, this may lead to increasing oxygen maintained in the vessel [8, 35–37]. Finally, others propose during diabetic disease, glycosylated hemoglobin has a higher affinity for oxygen, preventing these tightly bound molecules from readily diffusing into adjacent tissues [38–40]. Our study cannot ascertain by which mechanism oxygen saturation is increasing in those with retinopathy. This question may be better answered through longitudinal studies following subjects without retinopathy who transition into progressive grades of retinopathy as measured through tissue oxygen saturation and retinal imaging and blood flow measurements. However, our study has shown a structure/function change can be measured and captured at the capillary level as quantified by the OCTA and the oximeter.

This work also evaluated the relationship between function and structure in the macula as measured by oximetry and OCTA. Previous work has shown that vessel density measured by OCTA decreases with diabetes [41–43]. These vascular density changes can be attributed to chronic hypoxia and hyperglycemia, leading to impairment of vascular autoregulation [43]. Vascular remodeling and non-profusion contribute to capillary dropout as well [44]. In our study group, we found that these vascular changes correlate with oxygen saturation measurements, confirming a structure-function relationship within the macula in diabetic retinopathy. At this time, the causative relationship between retinal oxygen saturation and microvascular density is unknown. However, many studies have suggested that capillary occlusion and dropout from compromised endothelial vascular cells result in hypoxia, leading to increased retinal oxygen saturation demand [45–48].

In subjects with type 2 diabetes, oxygen saturation increases with duration of disease. This is in agreement with established literature showing duration of disease as a strong predictor of DR [5, 30], but we suggest that this is true at the capillary level as well. As duration increases, metabolic, inflammatory and oxidative stress accumulates within the retinal vasculature [2, 3]. Changes in oxygen saturation act as one of the metabolic stressors that increases the likelihood of retinopathy over time. This is strengthened by our previously discussed finding of those with DMR showing significantly higher retinal oxygen saturation compared to controls and DMnR. Many studies have shown once retinopathy is reached, increases in oxygen saturation are observed; [8, 29, 32] however, there are variabilities seen in oxygen saturation values once the retina reaches proliferation. Some studies show that oxygen saturation declines at the stage of proliferation; others have shown increases with proliferation [3, 6, 7, 9]. For our group of 8 subjects with severe to proliferative diabetic retinopathy, a wide range of oxygen saturation was observed. We considered whether visible signs of local retinal ischemia may be correlated to this variety of oximetry measures. Therefore, we also evaluated the number of cotton wool spots in each photo for potential answers in this small sample. These areas represent regions of axoplasmic accumulation, typically adjacent to arteriole occlusions in the superficial capillaries [49]. However, no relationship was found between cotton wool spots and oxygen saturation. At this time, the etiology of the large variation in oxygen saturation within those with severe NPDR and PDR is unknown. We suspect that it is likely these variations are due to a mixed mechanism and may include that of other systemic comorbidities such as hypertension. It is also possible changes in vessel dilation or the inclusion of larger retinal vessels in this sub-sample and in those with and without retinopathy could influence the structure-function relationship throughout the diabetes disease process. More work evaluating these vessel differences at different stages of retinal health would be needed to ascertain this influence.

There were limitations to this study. First, our sample of subjects with severe NPDR and PDR is small and was difficult to make any conclusions as to why there is such high variability in oxygen saturation in this group. We were unable to recruit additional subjects within this group as center-involving macular edema and vitreous hemorrhage are common complications of PDR that prevent usable oximetry data from being acquired. Future studies using retinal oximetry in larger populations of severe NPDR and PDR subjects will help to better understand those at risk for DR progression and may also help better understand response to treatment in these patient populations. A larger sample size will be needed of these subjects for further analyses of oxygen saturation and other ocular factors. Additionally, as this was a cross sectional study, subjects could only be compared across groups. A longitudinal study will be needed to evaluate pairwise comparisons through retinopathy progression. Future studies are thus needed to assess the relationship between oxygen saturation changes in diabetes and other comorbidities. Last, we could only evaluate a small retinal area in this study with 4 measures of 400 microns each. More retinal areas and other OCTA metrics derived from the Zeiss angioplex software could add to this work in the future.

## Conclusion

To our knowledge this is the first comparative study to evaluate macular vessel density and local oxygen saturation within the microvasculature between those with progressive stages of diabetic retinopathy and controls. This study links capillary bed deficits and oxygen saturation changes in the macula at the site of nutrient and oxygen exchange for those with diabetic retinopathy, suggesting that this structure-function relationship is quantifiable and similar in nature to that documented in larger diabetic retinal vasculature. However, these changes were not observed in DMnR subjects which may reflect variation in retinal function not captured by our testing parameters due to a wide range in disease severity and treatment compliance. Future longitudinal studies are needed to follow each of these groups over time to better assess the nuances in this structure-function relationship as subjects progress or revert in diabetic disease and retinopathy grades over time.

## Data Availability

The datasets used and/or analyzed during the current study are available from the corresponding author on reasonable request.

## Abbreviations

NPDR: Nonproliferative diabetic retinopathy
FAZ: Foveal avascular zone
OCTA: Optical coherence tomography angiography
SCP: Superficial capillary plexus
DMnR: Diabetes without retinopathy
DMR: Diabetes with retinopathy
DM: Diabetes
DR: Diabetic retinopathy
HbA1c: Hemoglobin A1c
PDR: Proliferative diabetic retinopathy
ETDRS: Early treatment diabetic retinopathy study
